# Effects of Pandemic on Feelings of Depression in Italy: The Role of Age, Gender, and Individual Experiences During the First Lockdown

**DOI:** 10.3389/fpsyg.2021.660628

**Published:** 2021-06-14

**Authors:** Bruno Arpino, Marta Pasqualini

**Affiliations:** ^1^Department of Statistics, Computer Science, Applications, University of Florence, Firenze, Italy; ^2^Observatoire Sociologique du Changement (OSC), Sciences Po, Paris, France

**Keywords:** coronavirus disease 2019, depressive feelings, life-course, gender, age, Italy

## Abstract

The restrictions to physical contacts that have been imposed in different countries to deal with the coronavirus disease 2019 (COVID-19) pandemic may have contributed to an increase in feelings of depression on top of other negative consequences of the pandemic. This study examines the consequences of the COVID-19 pandemic on feelings of depression using original data collected in Italy between April 14 and 24, 2020. Quota sampling (*N* = 3,026) was performed to target the population of 18+ and, together with post-stratification weights, permitted obtaining a representative sample of the Italian population with respect to key sociodemographic factors. We find that 47% of the respondents have increased depressive feelings during the Covid-19 lockdown. Adopting a life-course perspective, we revealed great heterogeneity in feelings of depression consequences by age, gender, and difficulties experienced during the first national lockdown. Identifying groups of population at higher risk of suffering from feelings of depression as a result of COVID-19 is crucial to limit indirect long-term consequences of the pandemic.

## Introduction

The health and economic crisis generated by the coronavirus disease 2019 (COVID-19) pandemic is unprecedented in recent human history. To this day, most scientific efforts have been dedicated to the study of the direct consequences of the pandemic on mortality and morbidity, especially among the oldest groups of the population (e.g., Drefahl et al., 2020), although studies focusing on consequences in other domains have been increasing as shown by Luppi et al. (2020) on fertility plans and Churchill (2020) on employment. Apart from physical health, the COVID-19 pandemic has affected psychological well-being. Previous studies have documented increased levels of depression (Arpino et al., 2020a; Mazza et al., 2020; Roma et al., 2020), stress (Mazza et al., 2020; Roma et al., 2020), loneliness (Orgilés et al., 2020), and anxiety (Mazza et al., 2020) during the lockdown period (for review Serafini et al., 2020 and Xiong et al., 2020). In fact, although necessary non-pharmaceutical measures implemented to fight the spread of COVID-19 have had and will likely produce profound and enduring consequences on the life of people (e.g., income and employment and interpersonal relationships), which, in turn, affect the psychological well-being of an individual.

Previous research has identified several factors associated with worsened psychological well-being during the lockdown. For example, studies have found that women and younger individuals have particularly suffered in terms of increased anxiety, depression, and stress (Mazza et al., 2020; Roma et al., 2020). However, age and gender have been usually analyzed as separate independent factors (e.g., Mazza et al., 2020; Pieh et al., 2020; Roma et al., 2020), and intersectionality among them has been overlooked. By adopting a life-course approach (Elder, 1994), this study contributes to growing literature by examining combined heterogeneities in the consequences of lockdown on self-reported changes in feelings of depression by age and gender among Italians aged 18+ during the first nationwide lockdown. Indeed, a life-course approach to the study on psychological consequences of the pandemic is crucial because individuals of all ages have been strongly affected by the pandemic, although differently and for different reasons (Settersten et al., 2020). Within the life-course approach, the impact of any event on individuals needs a time-based perspective, since the magnitude of effects likely depends on the age of an individual, which makes people more or less likely to be exposed to events, and it also influences their reaction to them (Settersten et al., 2020). Thus, for a better understanding of pandemic effects on the feelings of depression of individuals, it is crucial to look at age-differentiated phenomena that occurred during the lockdown. The pandemic and the restrictions implemented to fight it have indeed created a variety of stressors that are more or less likely to apply to an individual depending on their age. The youngest individuals have been affected, for example, by school and university closures and subsequent distance learning re-organization (Champeaux et al., 2020), resulting in negative psychological consequences (Liu et al., 2020; Spinelli et al., 2020). Many adults have been affected in terms of their income and employment (Churchill, 2020) or relationships, especially within the household because of more time spent at home (Balzarini et al., 2020; Bellani and Vignoli, 2020). Precarious work and poor relationship with partner or other people are known risk factors for depression (Paul and Moser, 2009; Teo et al., 2013). Thus, negative consequences of the lockdown in these areas are additional factors that have likely increased feelings of depression.

The COVID-19 pandemic may have an impact on psychological well-being because of bereavement for COVID-related deaths of close relatives or friends. A large number of individuals may have lost their partner or parent (Verdery et al., 2020), and, apart from death, the pandemic also brings with it worries of contagion of loved ones. However, a recent study has found that being in contact with an infected person had no effect on the psychological and emotional states of individuals (Kopilaš et al., 2021). Bereavement and worries of contagion represent transversal consequences of the pandemic to all life-course stages; however, their prevalence may vary by age, and they may also impact differently on individuals of different ages. Since COVID-19 containment measures also affected sport-related activities, home workout was the only option to play sports and stay active during the pandemic. Thus, another evident cross-age consequence of the lockdown and restrictive measures was reduced physical activity (Maugeri et al., 2020). Studies have found that the psychological well-being of individuals who reduced their level of physical exercise during the lockdown has been negatively affected (Callow et al., 2020; Maugeri et al., 2020; Pieh et al., 2020).

The life-course perspective also emphasizes that the effects of events experienced by individuals vary by their structural roles within the society. In particular, the consequences of the pandemic and containment measures are likely to be gendered because all the events that have been mentioned above may entail gendered dynamics. For example, more time spent at home has implied a re-organization of family work with greater burden on the shoulders of women, at least in more traditional contexts like Italy (Del Boca et al., 2020). More women seem to have lost their job because of lockdowns and physical distancing measures (Churchill, 2020). Also, the higher number of deaths for men from COVID-19 (Nasiri et al., 2020) implies that women are more likely to suffer from bereavement due to the death of their partner.

Given the above discussion, we examine the consequences of COVID-19 pandemic on feelings of depression in Italy according to three dimensions: age, gender, and type of negative events experienced during the first nationwide lockdown. More specifically, we consider the effect on the self-reported increase in feelings of depression of having experienced any of the following: reduction in physical activity, worsened relationship with the partner, worsened relationship with other people, suffered income loss, job loss, difficulties with organizing work or study from home, death of a relative or friend due to COVID-19, or infection of a relative or friend. We focus on Italy as it has been the first country outside Asia to be severely hit by the pandemic. Italy was also the first among Western countries to implement a nationwide lockdown, which has been one of the strictest and longest worldwide (Plümper and Neumayer, 2020).

The contribution of this study to the growing literature on the psychological consequences of the pandemic is 2-fold. First, we examine a large set of specific events that Italians may have experienced during the first lockdown. Second, inspired by the life-course perspective, we examine the combined heterogeneities in the consequences of these negative experiences by age and gender.

## Materials and Methods

### Data

The data were drawn from an online survey implemented in April 2020 on population aged 18+. The survey has been carried out through the online market survey platform called Lucid, which offers the opportunity to purchase samples for survey research and produces high quality, representative samples (Coppock and McClellan, 2019). Although an online survey can lead to coverage bias, the representativeness has been guaranteed by defining the sample quotas based on gender, age, region of residence, and education. Performing quota sampling ensures that the final sample is nearly identically distributed as the country benchmark given by the statistics provided by the national statistical offices on the key socio-demographic factors listed above. Additionally, we have used post-stratification weights to adjust for small deviations in the sample from the benchmark population statistics.

The analytic sample consists of 3,026 individuals aged 18+ living in Italy and involved in the survey between the 14th and 24th of April, 2020. For more information on the survey, and to consult the full questionnaire employed, refer to Arpino et al. (2020b).

### Dependent Variable

The dependent variable is a measure of self-reported changes in depressive feelings during the lockdown as compared with the pre-pandemic period. More specifically, the respondents were asked whether they felt sad or depressed either “more,” “equally,” or “less” often than usual (or not depressed at all) during the COVID-19 lockdown. This item allowed us to measure self-reported changes in feelings of depression in the absence of a pre-pandemic measure. The variable has been dichotomized, taking value 1 if a respondent reported to have felt sad or depressed more often than usual and 0 otherwise. Given the low number of individuals who reported feeling “less” depressed than before the lockdown (*N* = 115), when we tried dropping them from the analyses, the results were unchanged compared with those reported below.

### Explanatory Variables

The main independent variables are gender and age of respondents and events experienced during the pandemic. Gender is a dummy variable (1 = female, 0 = male). Age has been categorized and included in the analyses as a set of dummy variables for the age groups (18–25 = reference, 26–35; 36–49; 50–64; and 65+). A set of dummy variables accounted for whether the respondents have experienced (=1) or not (=0) each of the following during the lockdown: “reduction in physical activity;” “worsened relationship with partner;” “worsened relationship with other people;” “suffered income loss;” “lost job;” “difficulties with organizing work or study from home;” “death of a relative or friend due to COVID-19;” and “a relative or friend was infected.” Note that the respondents could report having experienced none of the previous circumstances, some of them, or even all of them.

### Control Variables

The multivariate analyses include a series of control variables that have been found to be related to feelings of depression or related measures in previous studies in the context of the pandemic (e.g., Arpino et al., 2020a; Callow et al., 2020; Mazza et al., 2020) and before it (e.g., Teo et al., 2013). Sociodemographic characteristics, such as subjective economic situation of the respondents (“living comfortably on present income” or “coping on present income” vs. “finding it difficult on present income” or “finding it very difficult on present income”) and the availability of kin (parents, children, and grandchildren) have been used as control variables. In addition, we controlled for the level of education (three levels based on the International Standard Classification of Education: “low” = below secondary education, “medium” = up to high school, and “high” = university education or above) and whether the respondents were or were not employed in the pre-COVID-19 period. Health was controlled by including two-health-related variables with regard to the period antecedent to the COVID-19 pandemic: self-perceived health of the respondents (0 = very good or good; 1 = fair, poor, or very poor) and any reported chronic disease (such as heart disease, hypertension, stroke, or cancer). An additional variable accounted for the severity level with which the region, where the respondents live was hit by the pandemic according to the tertiles of the distribution of Case-Fatality Rates (CFR) of COVID-19 at the regional level (NUTS-2).

### Statistical Methods

We first performed descriptive analyses on the main variables. Then, we used logistic regression models to examine the associations between the probability of increased perceived depressive feelings and gender, age, and difficulties experienced during the lockdown (Model 1). Then, we added to the model an interaction term between age and gender to test whether the two variables had a combined effect on the probability of increased depressive feelings (Model 2). Finally, we included three-way interactions between age, gender, and each of the potential issues experienced during the pandemic listed above. In other words, we tested whether age and gender moderated the effects on the worsened psychological well-being of reduction in physical activity, worsened relationship with the partner or with other people, having suffered income loss or loss of job, any difficulties with organizing work or study from home, death of a relative or friend due to COVID-19, or infection of a relative or friend (Model 3). To ease the interpretation of results, we calculated predicted probabilities by age and gender (based on estimates from Model 2) and by whether or not the respondents experienced each of the above-mentioned pandemic effects (based on Model 3).

## Results

Weighted descriptive statistics for the main variables of interest are reported in Table 1. During the lockdown, about 47% of Italians aged 18+ have felt sad or depressed more often than usual. The sample of respondents was equally composed of men and women (50%), and about 1/4 of them were aged 65+, while the youngest individuals (18–25 years old) constituted about 10% of the total sample. With regard to the difficulties experienced during the 1st month of lockdown, descriptive findings show that about 47% of the individuals have reported a reduction in physical activity. About 10–15% of the respondents reported worsened relationship with the partner and with other people. In addition, about 35% of the individuals have been affected by income loss and 6% have lost their job during the lockdown. Overall, 15% of the surveyed individuals have suffered from difficulties in organizing work/study at home. Finally, in this sample, the percentage of individuals who had at least a relative and/or friend infected or died from COVID-19 was 8 and 13%, respectively.

**Table 1:** Descriptive statistics.

Variables	%
**Dependent variable**	
Increased feeling of depression	47.25
**Independent variables**	
Age categories	
18–25	9.52
26–35	13.25
36–49	24.40
50–64	27.04
65+	25.79
**Gender**	
Female	50.00
Difficulties experienced during the lockdown	
Reduction in physical activity	46.60
Worsened relation with partner	9.04
Worsened relation with other people	14.20
Suffered income loss	34.62
Lost job	6.12
Difficulties with organizing work or study from home	15.16
Death of a relative or friend due to Coronavirus	7.59
A relative or friend was infected	13.05
Education: Low	16.06
Education: Medium	75.81
Education: High	8.13
Difficult or very difficult coping with income	34.50
Poor health	34.06
Chronic diseases	29.34
Employed	48.92
Any child(ren)	59.09
Any grandchild(ren)	22.55
Parents alive	50.34
In couple	60.23
Living with at least one coresident	88.87

*N* = 3,026. Post-stratification weights are used. Source: Intergen-coronavirus disease 2019 (COVID) online survey. Data were collected between April 14 and 24, 2020.

Full tables of estimated coefficients from the logistic regression models are reported in Supplementary Table A.1. In the following, we, mostly, focus on predicted probabilities from Models 2 and 3 described above. Regression results from Model 1 point to a higher probability of increased feelings of depression for women (*p* < 0.001; Supplementary Table A.1). As for age, apparently, there are no statistically significant effects, although estimates from Model 1 point to an age gradient with older individuals displaying lower probabilities of increased feelings of depression compared with the reference category (age 18–25; Supplementary Table A.1). However, and more interestingly, some age differences appear more clearly from the predicted probabilities based on Model 2, which adds interactions between age and gender (Table 2).

**Table 2:** Predicted probabilities of increased feeling of depression by age and gender (CI for 5% pairwise comparisons).

Gender	Age				
	18–25	26–35	36–49	50–64	65+
Men	0.41 (0.35; 0.47)	0.49 (0.44; 0.55)	0.40 (0.36; 0.44)	0.39 (0.35; 0.43)	0.35 (0.30; 0.39)
Women	0.53 (0.47; 0.59)	0.54 (0.49; 0.58)	0.56 (0.52; 0.60)	0.51 (0.48; 0.55)	0.54 (0.49; 0.59)

*N* = 3,026. Post-stratification weights are used. Non-overlapping CIs indicate that the corresponding predicted probabilities are statistically significant with difference at the 5% level. Source: Intergen-COVID online survey. Data were collected between April 14 and 24, 2020.

Predicted probabilities, as shown in Tables 2 and 3, are presented together with CIs for pairwise comparisons. These intervals are centered on the predictions and have lengths equal to 2 × 1.39 × SEs. As shown by Goldstein and Healy (1995), this is necessary in order to have an average level of 5% significance for pairwise comparisons. In other words, in this way, we can compare any pair of CIs and interpret an overlap between them as no statistically significant difference between the corresponding predictions, whereas non-overlap reflects that they are at the 5%.

**Table 3:** Predicted probabilities of increased feeling of depression by age, gender, and difficulties experienced during the lockdown (CI for 5% pairwise comparisons).

Difficulties experienced during the lockdown	Age				
	18–25	26–35	36–49	50–64	65+
**Reduction in physical activity**					
Yes					
Men	0.50 (0.40; 0.60)	0.59 (0.50; 0.67)	0.43 (0.38; 0.49)	0.41 (0.36; 0.45)	0.46 (0.40; 0.52)
Women	0.47 (0.38; 0.55)	0.65 (0.59; 0.72)	0.64 (0.59; 0.69)	0.58 (0.53; 0.64)	0.62 (0.55; 0.70)
No					
Men	0.34 (0.27; 0.41)	0.42 (0.35; 0.49)	0.38 (0.33; 0.43)	0.39 (0.34; 0.44)	0.25 (0.19; 0.31)
Women	0.57 (0.50; 0.65)	0.45 (0.39; 0.51)	0.49 (0.44; 0.54)	0.45 (0.40; 0.50)	0.47 (0.39; 0.54)
**Worsened relation with partner**					
Yes					
Men	0.28 (0.09; 0.48)	0.73 (0.59; 0.87)	0.77 (0.66; 0.88)	0.70 (0.59; 0.81)	0.71 (0.57; 0.85)
Women	0.76 (0.65; 0.88)	0.66 (0.55; 0.76)	0.81 (0.72; 0.90)	0.70 (0.58; 0.81)	0.41 (0.19; 0.64)
No					
Men	0.43 (0.37; 0.49)	0.47 (0.41; 0.53)	0.37 (0.33; 0.41)	0.36 (0.32; 0.40)	0.31 (0.26; 0.36)
Women	0.51 (0.44; 0.58)	0.53 (0.49; 0.58)	0.54 (0.50; 0.58)	0.50 (0.46; 0.53)	0.53 (0.48; 0.59)
**Worsened relation with other people**					
Yes					
Men	0.53 (0.41; 0.66)	0.54 (0.42; 0.66)	0.57 (0.48; 0.66)	0.53 (0.55; 0.63)	0.47 (0.34; 0.60)
Women	0.59 (0.45; 0.74)	0.66 (0.56; 0.76)	0.62 (0.52; 0.71)	0.58 (0.48; 0.69)	0.55 (0.35; 0.75)
No					
Men	0.38 (0.32; 0.45)	0.49 (0.43; 0.55)	0.36 (0.32; 0.41)	0.37 (0.33; 0.41)	0.33 (0.28; 0.37)
Women	0.53 (0.46; 0.59)	0.52 (0.47; 0.57)	0.55 (0.51; 0.59)	0.50 (0.46; 0.54)	0.53 (0.48; 0.59)
**Suffered income loss**					
Yes					
Men	0.42 (0.28; 0.55)	0.54 (0.46; 0.63)	0.48 (0.42; 0.54)	0.43 (0.37; 0.48)	0.47 (0.37; 0.56)
Women	0.52 (0.46; 0.58)	0.52 (0.46; 0.58)	0.61 (0.56; 0.66)	0.52 (0.46; 0.57)	0.56 (0.45; 0.67)
No					
Men	0.39 (0.33; 0.46)	0.47 (0.40; 0.54)	0.34 (0.30; 0.39)	0.37 (0.33; 0.42)	0.31 (0.26; 0.36)
Women	0.55 (0.43; 0.67)	0.58 (0.52; 0.64)	0.53 (0.49; 0.58)	0.51 (0.47; 0.56)	0.52 (0.46; 0.59)
**Lost job**					
Yes					
Men	0.48 (0.26; 0.70)	0.53 (0.36; 0.70)	0.45 (0.33; 0.57)	0.51 (0.38; 0.65)	0.37 (0.10; 0.64)
Women	0.51 (0.32; 0.70)	0.60 (0.47; 0.73)	0.70 (0.58; 0.81)	0.42 (0.29; 0.55)	0.20 (−0.00; 0.41)
No					
Men	0.40 (0.34; 0.46)	0.49 (0.43; 0.55)	0.39 (0.36; 0.43)	0.38 (0.34; 0.42)	0.35 (0.30; 0.39)
Women	0.54 (0.47; 0.60)	0.53 (0.48; 0.58)	0.55 (0.51; 0.58)	0.52 (0.48; 0.55)	0.55 (0.50; 0.60)
**Difficulties with organizing work or study from home**					
Yes					
Men	0.54 (0.43; 0.65)	0.72 (0.60; 0.84)	0.40 (0.31; 0.49)	0.50 (0.40; 0.59)	0.42 (0.24; 0.61)
Women	0.65 (0.56; 0.74)	0.67 (0.57; 0.76)	0.60 (0.52; 0.68)	0.81 (0.73; 0.89)	0.63 (0.33; 0.93)
No					
Men	0.38 (0.31; 0.45)	0.45 (0.39; 0.51)	0.40 (0.36; 0.45)	0.37 (0.33; 0.41)	0.33 (0.28; 0.37)
Women	0.52 (0.44; 0.59)	0.52 (0.47; 0.57)	0.56 (0.52; 0.59)	0.47 (0.44; 0.51)	0.52 (0.46; 0.58)
**Death of a relative or friend due to Coronavirus**					
Yes					
Men	0.64 (0.41; 0.88)	0.61 (0.34; 0.87)	0.36 (0.23; 0.50)	0.19 (0.10; 0.29)	0.35 (0.21; 0.48)
Women	0.62 (0.43; 0.82)	0.60 (0.44; 0.76)	0.63 (0.45; 0.81)	0.60 (0.49; 0.72)	0.81 (0.66; 0.96)
No					
Men	0.40 (0.34; 0.46)	0.49 (0.43; 0.54)	0.40 (0.36; 0.44)	0.41 (0.37; 0.45)	0.35 (0.31; 0.40)
Women	0.52 (0.46; 0.59)	0.53 (0.49; 0.58)	0.55 (0.51; 0.59)	0.50 (0.47; 0.54)	0.51 (0.46; 0.57)
**A relative or friend was infected**					
Yes					
Men	0.35 (0.19; 0.50)	0.62 (0.49; 0.76)	0.45 (0.34; 0.56)	0.39 (0.28; 0.49)	0.28 (0.17; 0.39)
Women	0.62 (0.46; 0.79)	0.51 (0.41; 0.62)	0.67 (0.59; 0.75)	0.53 (0.44; 0.63)	0.67 (0.53; 0.82)
No					
Men	0.41 (0.35; 0.48)	0.47 (0.41; 0.53)	0.39 (0.35; 0.43)	0.39 (0.35; 0.43)	0.35 (0.31; 0.40)
Women	0.52 (0.45; 0.58)	0.55 (0.50; 0.59)	0.54 (0.50; 0.58)	0.51 (0.47; 0.55)	0.53 (0.47; 0.58)

*N* = 3,026. Post-stratification weights are used. Non-overlapping CIs indicate that the corresponding predicted probabilities are statistically significant with difference at the 5% level. Source: Intergen-COVID online survey. Data were collected between April 14 and 24, 2020.

Table 2 displays predicted probabilities of increased feelings of depression by age and gender (Model 2) showing that women are considerably more likely to have experienced increased feelings of depression compared with men. In fact, predicted probabilities for women are between 12 and 19 percentage points higher than those of men, but gender differences are statistically significant (*p* < 0.05) only for the three oldest age groups considered (36+). As for age, we observe a gendered pattern. While predicted probabilities of increased feelings of depression do not substantially vary across age groups for women (and all of them are above 50%), younger men tend to report considerably higher predicted probabilities compared with the oldest ones. In particular, men aged 26–36 represent the group at highest risk of increased depressive feelings, with a predicted probability of 49%, which is between 10 and 14 percentage points higher (and also statistically different) than those for the oldest age group considered (50–64 and 65+, respectively). Overall, women and adult men aged 26–36 are those who seem to have suffered more during the lockdown.

As for the events experienced during the lockdown, the results from Models 1 and 2 are very similar and show that most of the considered circumstances are significantly associated with a higher probability of increased feelings of depression, with few exceptions (Supplementary Table A.1). However, interests of authors are in assessing whether these effects vary by age and gender.

Table 3 shows predicted probabilities of increased feelings of depression by difficulties experienced during the lockdown, age groups, and gender. As expected, different circumstances showed a meaningful association with increased feelings of depression during the lockdown for different subgroups of the population. Reduced physical activity significantly increased the probability of increased feelings of depression especially among women aged 26+ and men aged 65+ (*p* < 0.01). The effects are also significant from a substantive point of view, especially for the oldest individuals. For example, among men aged 65+, those who did not reduce their physical activities had a 25% probability of increased feelings of depression compared with a 46% probability for those who did experience a reduction in physical activity. Having experienced a deterioration in relationship quality with partner was associated with an increased probability of feelings of depression, and differences were statistically significant for almost all age and gender groups. Of note is that for two subgroups (men aged 18–25 and women aged 65+), CIs are rather large because of the low proportion of individuals with a partner within these subgroups. When statistically significant, the effect of worsened relationship with the partner is also substantial: worsened association with partner is associated with about 20 percentage points higher probability of increased feelings of depression. Worsened relationship with other people, instead, displays a statistically significant effect only among adult men aged 36–64. Gaps are also rather substantial, amounting to about 10 additional percentage points of increased feelings of depression for those who experienced poorer relationship quality compared with those who did not.

Turning to the economic consequences of the pandemic, although income loss was significantly associated with the probability of increased feeling of depression in the whole sample (Models 1 and 2, Supplementary Table A.1), when interacting it with age and gender, it has a significant effect only on two groups (men aged 36–49 and men aged 65+). Having experienced a job loss (this was the case only for about 6% of the sample, Table 1) was never statistically associated with increased depressive feelings, with the exception of women aged 36–49 who were considerably more at risk of losing their job and thus suffering psychologically from it (predicted probabilities: 70 and 55% for those who experienced job loss and who did not experience job loss, respectively, in this age group).

Difficulties in organizing work or study from home negatively affected feelings of depression, especially for younger individuals. In particular, men aged 26–35 who experienced these negative effects of the lockdown displayed a probability of increased feelings of depression of 72 vs. 45%, in case they were not affected in this respect.

Finally, as for the health consequences of the pandemic, the respondents who have experienced death of a relative or friend due to COVID-19 were, as expected, more likely to have felt sad or depressed more often than usual. However, the differences within age-gender subgroups are usually not statistically significant because of the small number of individuals in this study sample, who experienced this event. Exceptions are found among the oldest individuals, and, in particular, for women aged 65+, it is found that one of the highest predicted probability of increased feeling of depression across all cells if they experienced a COVID-related death of a relative or friend (81 vs. 51% if they did not experience the event). Knowing that a relative or friend had been infected with COVID-19 was not significantly associated with increased feelings of depression.

## Discussion

This study has adopted a life-course approach for assessing the feelings of depression among Italians aged 18+ during the early phase of the COVID-19 pandemic. More specifically, we have examined the probability of increased feelings of depression according to three dimensions: age, gender, and type of negative events experienced during the first national lockdown.

**Figure 1:**
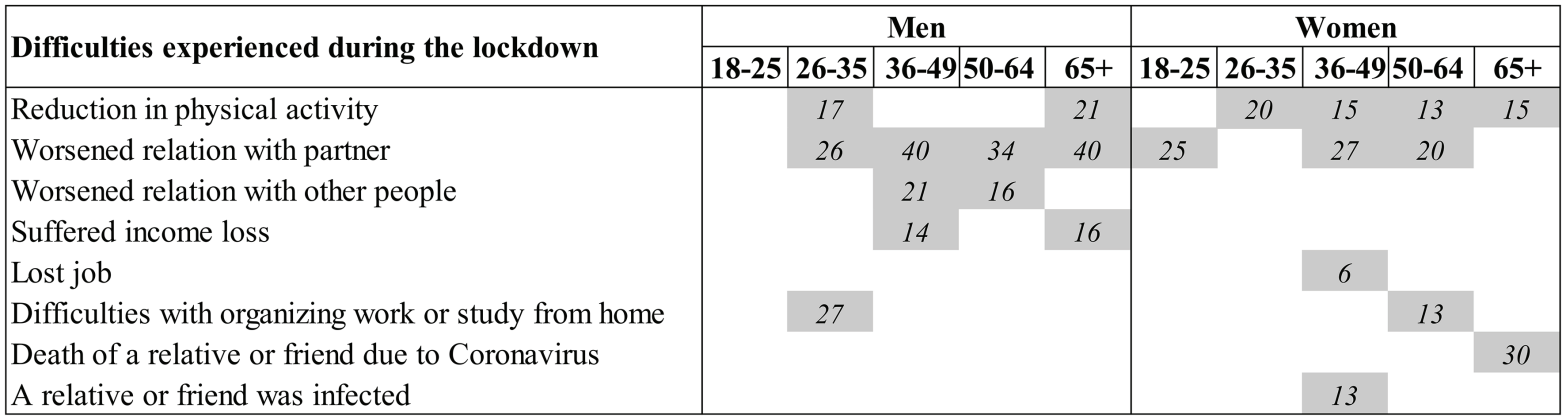
Visual summary of main results: statistically and substantively significant effects of difficulties experienced during the lockdown on the probability of increased feelings of depression by age and gender. Gray cells highlight statistically significant (at the 5% level) and substantively important associations. The numbers in the highlighted cells are the difference in the predicted probability of increased depression feelings for those who did and did not experience each event (percentage points).

Empirical findings, based on original data collected in April 2020, showed that the pandemic has created a variety of stressors whose effects strongly depend on the gender and age of individuals. In line with existing studies (e.g., Mazza et al., 2020), we found that women reported more frequently than men to have experienced increased feelings of depression. In addition, consistent with similar findings from Italy (e.g., Roma et al., 2020), younger respondents were generally more likely to have experienced increased feelings of depression. However, the results showed that this was especially the case for men. Finally, while all age and gender sub-groups suffered a considerable increase in their feelings of depression when compared with the pre-COVID period, we found a substantial degree of heterogeneity in the extent this was the case. Specifically, we examined this heterogeneity as a function of specific events experienced during the early phase of the COVID-19 pandemic. Figure 1 graphically summarizes these results.

Some of the consequences of the pandemic that we accounted for negatively affected feelings of depression among almost all demographic subgroups we considered. This was the case for worsened quality of partnership relationship and reduction of physical activity (especially for women). These findings are in line with those of some previous studies (e.g., Teo et al., 2013; Maugeri et al., 2020). Instead, other events experienced during the early stage of the pandemic had age- and/or gender-specific effects on feelings of depression (Figure 1). A group-specific effect was increased feelings of depression associated with worsened relationship with people other than the partner that emerged only for adult men aged 36–64. Although the data were not informative about this, we may speculate that this might be driven by relationships with coworkers. The youngest individuals (aged 18–35) suffered, especially, because of difficulties in organizing study and work from home. The only subgroup displaying a meaningful effect on increased feelings of depression due to the economic consequences of the lockdown and restrictive measures, i.e., income loss was constituted by adult men aged 36–54. Older people aged 50+ experienced more frequently than other groups increased feelings of depression due to the death of a relative or friend from COVID-19. Instead, and in line with previous findings (Kopilaš et al., 2021), this results did not provide evidence on the link between the contagion of relatives or friends with increased feelings of depression.

As shown in Figure 1, the associations between having experienced or not certain events during the pandemic and increased feelings of depression that we have found were not only significant from a statistical point of view, but they were also substantial, with differences in the predicted probability of increased feelings of depression ranging between 6 and 40 percentage points. The strongest effect was found for having experienced worsened relationship with partner among men aged 36–49 and 65+ (40 percentage points higher predicted probability of increased depression feelings among those who experience this change during the lockdown).

Overall, the economic consequences of the pandemic (income and job loss) were insignificant (both statistically and substantially) in this study, with few exceptions. This may be because of the fact that we conducted this study at a time, where the effects of the pandemic were not yet clear, and the duration of the pandemic was also uncertain, so some of the individuals who experienced economic shocks might have relatively positive expectations on a rapid solution of the health crisis and about the recovery of the general economic situation and their job and income status. This finding, however, may also relate to the relatively small number of individuals in this study sample, who experienced negative economic effects, especially job loss. Thus, this finding requires further investigation in future studies, especially with data collected at the following stages of the pandemic.

One limitation of this study is that the results may not generalize to the whole population, because the data are based on an online survey. However, online data collection was almost the only choice we had during the lockdown. Additionally, by quota sampling and post-stratification weights, we made the sample representative of the Italian 18+ population with respect to key sociodemographic variables. We could not explore some aspects because of the restrictions to the questionnaire length required by the online data collection mode. For example, we did not collect information on how work or study was organized at a distance. Also, worsened relationship quality was distinguished only if related to the partner or other people, but more detailed differentiation, e.g., between children, other relatives, friends, and co-workers, is an interesting topic for future studies. Finally, this study uses a self-reported measure of increased feelings of depression during the lockdown, instead of a validated scale of depression. The measure used in this study might be subjected to several sources of bias, such as social desirability bias.

Despite these limitations, this study contributes to the identification of subgroups of population at higher risk of worsened psychological well-being due to the COVID-19 pandemic, which is crucial to limit long-term consequences of this pandemic. The results point to the fact that although all individuals may have experienced negative effects on psychological well-being, the extent of these effects greatly varies across different subgroups. Combinations of several dimensions, such as age and gender, are crucial in identifying individuals who suffered the most. Future studies may consider further relevant dimensions, such as socioeconomic status.

## Data Availability Statement

The datasets presented in this article are not readily available because the individual data cannot be provided but descriptive statistics and questionnaire are available at a dedicated website: https://sites.google.com/unifi.it/intergen-covid. Requests to access the datasets should be directed to https://sites.google.com/unifi.it/intergen-covid.

## Ethics Statement

Ethical review and approval was not required for the study on human participants in accordance with the local legislation and institutional requirements. The patients/participants provided their written informed consent to participate in this study.

## Author Contributions

All authors listed have made a substantial, direct and intellectual contribution to the work, and approved it for publication.

### Conflict of Interest

The authors declare that the research was conducted in the absence of any commercial or financial relationships that could be construed as a potential conflict of interest.
