# Supplementary material for: Effects of Pandemic on Feelings of Depression in Italy: The Role of Age, Gender, and Individual Experiences During the First Lockdown
Source: Front Psychol. 2021 Jun 14;12:660628. doi: 10.3389/fpsyg.2021.660628 (PMC8236577; doi:10.3389/fpsyg.2021.660628)
Supplement: Supplementary file 1 [file Table_1.DOCX]

Supplementary Material

# Table A.1 Associations between Age, Gender, Difficulties experienced during the Lockdown and Worsen Depression (Logistic regression Coefficients and Standard Error)

| VARIABLES | M1 | M2 | M3 | | | | | | | |
| --- | --- | --- | --- | --- | --- | --- | --- | --- | --- | --- |
|  |  |  |  |  |  |  |  |  |  |  |
| Age: 26-35 (ref. 18-25) | 0.220 | 0.389 | 0.377 | 0.202 | 0.454 | 0.346 | 0.398 | 0.301 | 0.404 | 0.254 |
|  | (0.182) | (0.257) | (0.329) | (0.260) | (0.286) | (0.302) | (0.269) | (0.294) | (0.262) | (0.272) |
| 36-49 | 0.0405 | -0.0413 | 0.177 | -0.265 | -0.0926 | -0.219 | -0.0393 | 0.111 | 0.00333 | -0.103 |
|  | (0.172) | (0.233) | (0.294) | (0.235) | (0.262) | (0.270) | (0.244) | (0.262) | (0.238) | (0.243) |
| 50-64 | -0.0811 | -0.0781 | 0.248 | -0.290 | -0.0805 | -0.0850 | -0.0966 | -0.0425 | 0.0634 | -0.107 |
|  | (0.181) | (0.240) | (0.306) | (0.242) | (0.267) | (0.274) | (0.250) | (0.269) | (0.243) | (0.249) |
| 65+ | -0.105 | -0.288 | -0.455 | -0.536* | -0.264 | -0.397 | -0.267 | -0.263 | -0.213 | -0.284 |
|  | (0.216) | (0.273) | (0.355) | (0.276) | (0.294) | (0.295) | (0.279) | (0.297) | (0.277) | (0.281) |
| Gender: Female | 0.626*** | 0.558** | 1.03*** | 0.363 | 0.623** | 0.565** | 0.600** | 0.598* | 0.572** | 0.466* |
|  | (0.090) | (0.260) | (0.327) | (0.268) | (0.288) | (0.287) | (0.270) | (0.315) | (0.269) | (0.274) |
| Reduction in physical activity | 0.538*** | 0.533*** | 0.718* | 0.537*** | 0.539*** | 0.533*** | 0.532*** | 0.527*** | 0.549*** | 0.534*** |
|  | (0.092) | (0.092) | (0.386) | (0.092) | (0.093) | (0.093) | (0.092) | (0.092) | (0.092) | (0.092) |
| Worsened relation with partner | 1.039*** | 1.051*** | 1.062*** | -0.674 | 1.050*** | 1.045*** | 1.048*** | 1.052*** | 1.044*** | 1.048*** |
|  | (0.169) | (0.171) | (0.174) | (0.766) | (0.166) | (0.170) | (0.172) | (0.172) | (0.170) | (0.170) |
| Worsened relation with other people | 0.522*** | 0.529*** | 0.502*** | 0.502*** | 0.662 | 0.524*** | 0.530*** | 0.553*** | 0.526*** | 0.524*** |
|  | (0.132) | (0.132) | (0.132) | (0.129) | (0.441) | (0.133) | (0.132) | (0.132) | (0.132) | (0.132) |
| Suffered income loss | 0.309*** | 0.307*** | 0.298*** | 0.316*** | 0.299*** | 0.112 | 0.326*** | 0.321*** | 0.312*** | 0.305*** |
|  | (0.101) | (0.101) | (0.101) | (0.102) | (0.101) | (0.483) | (0.101) | (0.101) | (0.102) | (0.101) |
| Lost job | 0.153 | 0.152 | 0.163 | 0.141 | 0.157 | 0.180 | 0.366 | 0.127 | 0.122 | 0.153 |
|  | (0.190) | (0.190) | (0.187) | (0.194) | (0.190) | (0.189) | (0.735) | (0.183) | (0.199) | (0.190) |
| Difficulties with organizing work or study from home | 0.594*** | 0.586*** | 0.594*** | 0.588*** | 0.580*** | 0.575*** | 0.622*** | 0.700* | 0.569*** | 0.591*** |
|  | (0.131) | (0.130) | (0.130) | (0.130) | (0.131) | (0.131) | (0.127) | (0.406) | (0.134) | (0.131) |
|  |  |  |  |  |  |  |  |  |  |  |
| Death of a relative or friend due to Coronavirus | 0.292 | 0.298 | 0.305 | 0.296 | 0.302 | 0.301 | 0.345 | 0.308 | 1.104 | 0.315 |
|  | (0.236) | (0.232) | (0.233) | (0.232) | (0.230) | (0.231) | (0.223) | (0.229) | (0.826) | (0.240) |
| A relative or friend was infected | 0.212 | 0.213 | 0.210 | 0.221 | 0.213 | 0.228 | 0.195 | 0.195 | 0.247* | -0.322 |
|  | (0.147) | (0.145) | (0.145) | (0.145) | (0.144) | (0.145) | (0.144) | (0.147) | (0.142) | (0.590) |
| Female* 26-35 (ref. 18-25) |  | -0.363 | -0.895** | -0.106 | -0.476 | -0.361 | -0.423 | -0.292 | -0.374 | -0.131 |
|  |  | (0.338) | (0.435) | (0.352) | (0.371) | (0.407) | (0.353) | (0.397) | (0.348) | (0.362) |
| Female* 36-49 |  | 0.159 | -0.545 | 0.379 | 0.204 | 0.276 | 0.0797 | 0.0695 | 0.116 | 0.205 |
|  |  | (0.307) | (0.396) | (0.319) | (0.339) | (0.361) | (0.319) | (0.361) | (0.316) | (0.326) |
| Female* 50-64 |  | -0.0153 | -0.777* | 0.225 | -0.0282 | 0.0646 | 0.0104 | -0.142 | -0.158 | 0.0656 |
|  |  | (0.308) | (0.400) | (0.319) | (0.339) | (0.360) | (0.320) | (0.361) | (0.318) | (0.327) |
| Female* 65+ |  | 0.322 | 0.00822 | 0.631* | 0.301 | 0.420 | 0.322 | 0.282 | 0.160 | 0.318 |
|  |  | (0.340) | (0.459) | (0.350) | (0.368) | (0.379) | (0.348) | (0.385) | (0.346) | (0.358) |
| Female *Reduction in physical activity |  |  | -1.171** |  |  |  |  |  |  |  |
|  |  |  | (0.519) |  |  |  |  |  |  |  |
| Age: 26-35 (ref. 18-25) *Reduction in physical activity |  |  | 0.0141 |  |  |  |  |  |  |  |
|  |  |  | (0.521) |  |  |  |  |  |  |  |
| 36-49 * Reduction in physical activity |  |  | -0.461 |  |  |  |  |  |  |  |
|  |  |  | (0.452) |  |  |  |  |  |  |  |
| 50-64 * Reduction in physical activity |  |  | -0.648 |  |  |  |  |  |  |  |
|  |  |  | (0.453) |  |  |  |  |  |  |  |
| 65+ * Reduction in physical activity |  |  | 0.285 |  |  |  |  |  |  |  |
|  |  |  | (0.475) |  |  |  |  |  |  |  |
| Female* 26-35 (ref. 18-25) *Reduction in physical activity |  |  | 1.328* |  |  |  |  |  |  |  |
|  |  |  | (0.688) |  |  |  |  |  |  |  |
| Female* 36-49 * Reduction in physical activity |  |  | 1.604*** |  |  |  |  |  |  |  |
|  |  |  | (0.613) |  |  |  |  |  |  |  |
| Female* 50-64 * Reduction in physical activity |  |  | 1.683*** |  |  |  |  |  |  |  |
|  |  |  | (0.615) |  |  |  |  |  |  |  |
| Female* 65+ * Reduction in physical activity |  |  | 0.836 |  |  |  |  |  |  |  |
|  |  |  | (0.678) |  |  |  |  |  |  |  |
| Female *Worsened relation with partner |  |  |  | 1.872** |  |  |  |  |  |  |
|  |  |  |  | (0.929) |  |  |  |  |  |  |
| Age: 26-35 (ref. 18-25) *Worsened relation with partner |  |  |  | 1.865* |  |  |  |  |  |  |
|  |  |  |  | (0.961) |  |  |  |  |  |  |
| 36-49 * Worsened relation with partner |  |  |  | 2.545*** |  |  |  |  |  |  |
|  |  |  |  | (0.916) |  |  |  |  |  |  |
| 50-64 * Worsened relation with partner |  |  |  | 2.189** |  |  |  |  |  |  |
|  |  |  |  | (0.878) |  |  |  |  |  |  |
| 65+ * Worsened relation with partner |  |  |  | 2.497*** |  |  |  |  |  |  |
|  |  |  |  | (0.937) |  |  |  |  |  |  |
| Female* 26-35 (ref. 18-25) *Worsened relation with partner |  |  |  | -2.510** |  |  |  |  |  |  |
|  |  |  |  | (1.163) |  |  |  |  |  |  |
| Female* 36-49 * Worsened relation with partner |  |  |  | -2.341** |  |  |  |  |  |  |
|  |  |  |  | (1.148) |  |  |  |  |  |  |
| Female* 50-64 * Worsened relation with partner |  |  |  | -2.473** |  |  |  |  |  |  |
|  |  |  |  | (1.113) |  |  |  |  |  |  |
| Female* 65+ * Worsened relation with partner |  |  |  | -4.205*** |  |  |  |  |  |  |
|  |  |  |  | (1.308) |  |  |  |  |  |  |
| Female *Worsened relation with other people |  |  |  |  | -0.359 |  |  |  |  |  |
|  |  |  |  |  | (0.676) |  |  |  |  |  |
| Age: 26-35 (ref. 18-25) *Worsened relation with other |  |  |  |  | -0.420 |  |  |  |  |  |
|  |  |  |  |  | (0.617) |  |  |  |  |  |
| 36-49 * Worsened relation with other |  |  |  |  | 0.243 |  |  |  |  |  |
|  |  |  |  |  | (0.551) |  |  |  |  |  |
| 50-64 * Worsened relation with other |  |  |  |  | 0.0757 |  |  |  |  |  |
|  |  |  |  |  | (0.548) |  |  |  |  |  |
| 65+ * Worsened relation with other |  |  |  |  | -0.0286 |  |  |  |  |  |
|  |  |  |  |  | (0.614) |  |  |  |  |  |
| Female* 26-35 (ref. 18-25) *Worsened relation with other |  |  |  |  | 0.734 |  |  |  |  |  |
|  |  |  |  |  | (0.886) |  |  |  |  |  |
| Female* 36-49 * Worsened relation with other |  |  |  |  | -0.248 |  |  |  |  |  |
|  |  |  |  |  | (0.823) |  |  |  |  |  |
| Female* 50-64 * Worsened relation with other |  |  |  |  | -0.0144 |  |  |  |  |  |
|  |  |  |  |  | (0.834) |  |  |  |  |  |
| Female* 65+ * Worsened relation with other |  |  |  |  | -0.203 |  |  |  |  |  |
|  |  |  |  |  | (1.047) |  |  |  |  |  |
| Female *Suffered income loss |  |  |  |  |  | 0.0287 |  |  |  |  |
|  |  |  |  |  |  | (0.652) |  |  |  |  |
| Age: 26-35 (ref. 18-25) *Suffered income loss |  |  |  |  |  | 0.207 |  |  |  |  |
|  |  |  |  |  |  | (0.592) |  |  |  |  |
| 36-49 *Suffered income loss |  |  |  |  |  | 0.515 |  |  |  |  |
|  |  |  |  |  |  | (0.540) |  |  |  |  |
| 50-64 *Suffered income loss |  |  |  |  |  | 0.134 |  |  |  |  |
|  |  |  |  |  |  | (0.537) |  |  |  |  |
| 65+ * Suffered income loss |  |  |  |  |  | 0.628 |  |  |  |  |
|  |  |  |  |  |  | (0.590) |  |  |  |  |
| Female* 26-35 (ref. 18-25) *Suffered income loss |  |  |  |  |  | -0.0533 |  |  |  |  |
|  |  |  |  |  |  | (0.787) |  |  |  |  |
| Female* 36-49 *Suffered income loss |  |  |  |  |  | -0.313 |  |  |  |  |
|  |  |  |  |  |  | (0.733) |  |  |  |  |
| Female* 50-64 *Suffered income loss |  |  |  |  |  | -0.261 |  |  |  |  |
|  |  |  |  |  |  | (0.730) |  |  |  |  |
| Female* 65+ * Suffered income loss |  |  |  |  |  | -0.606 |  |  |  |  |
|  |  |  |  |  |  | (0.843) |  |  |  |  |
| Female *Lost job |  |  |  |  |  |  | -0.497 |  |  |  |
|  |  |  |  |  |  |  | (0.971) |  |  |  |
| Age: 26-35 (ref. 18-25) *Lost job |  |  |  |  |  |  | -0.203 |  |  |  |
|  |  |  |  |  |  |  | (0.924) |  |  |  |
| 36-49 *Lost job |  |  |  |  |  |  | -0.117 |  |  |  |
|  |  |  |  |  |  |  | (0.844) |  |  |  |
| 50-64 *Lost job |  |  |  |  |  |  | 0.223 |  |  |  |
|  |  |  |  |  |  |  | (0.867) |  |  |  |
| 65+ * Lost job |  |  |  |  |  |  | -0.249 |  |  |  |
|  |  |  |  |  |  |  | (1.199) |  |  |  |
| Female* 26-35 (ref. 18-25) *Lost job |  |  |  |  |  |  | 0.646 |  |  |  |
|  |  |  |  |  |  |  | (1.207) |  |  |  |
| Female* 36-49 *Lost job |  |  |  |  |  |  | 0.978 |  |  |  |
|  |  |  |  |  |  |  | (1.143) |  |  |  |
| Female* 50-64 *Lost job |  |  |  |  |  |  | -0.517 |  |  |  |
|  |  |  |  |  |  |  | (1.160) |  |  |  |
| Female* 65+ * Lost job |  |  |  |  |  |  | -1.388 |  |  |  |
|  |  |  |  |  |  |  | (1.717) |  |  |  |
| Female *Difficulties with organizing work or study from home |  |  |  |  |  |  |  | -0.110 |  |  |
|  |  |  |  |  |  |  |  | (0.555) |  |  |
| Age: 26-35 (ref. 18-25) *Difficulties with organizing work or study from home |  |  |  |  |  |  |  | 0.551 |  |  |
|  |  |  |  |  |  |  |  | (0.638) |  |  |
| 36-49 *Difficulties with organizing work or study from home |  |  |  |  |  |  |  | -0.720 |  |  |
|  |  |  |  |  |  |  |  | (0.510) |  |  |
| 50-64 *Difficulties with organizing work or study from home |  |  |  |  |  |  |  | -0.128 |  |  |
|  |  |  |  |  |  |  |  | (0.519) |  |  |
| 65+ * Difficulties with organizing work or study from home |  |  |  |  |  |  |  | -0.241 |  |  |
|  |  |  |  |  |  |  |  | (0.740) |  |  |
| Female* 26-35 (ref. 18-25) *Difficulties with organizing work or study from home |  |  |  |  |  |  |  | -0.447 |  |  |
|  |  |  |  |  |  |  |  | (0.825) |  |  |
| Female* 36-49 *Difficulties with organizing work or study from home |  |  |  |  |  |  |  | 0.328 |  |  |
|  |  |  |  |  |  |  |  | (0.697) |  |  |
| Female* 50-64 *Difficulties with organizing work or study from home |  |  |  |  |  |  |  | 1.246 |  |  |
|  |  |  |  |  |  |  |  | (0.789) |  |  |
| Female* 65+ * Difficulties with organizing work or study from home |  |  |  |  |  |  |  | 0.147 |  |  |
|  |  |  |  |  |  |  |  | (1.323) |  |  |
| Female *Death of a relative or friend due to Coronavirus |  |  |  |  |  |  |  |  | -0.663 |  |
|  |  |  |  |  |  |  |  |  | (1.065) |  |
| Age: 26-35 (ref. 18-25) *Death of a relative or friend due to Coronavirus |  |  |  |  |  |  |  |  | -0.564 |  |
|  |  |  |  |  |  |  |  |  | (1.223) |  |
| 36-49 *Death of a relative or friend due to Coronavirus |  |  |  |  |  |  |  |  | -1.256 |  |
|  |  |  |  |  |  |  |  |  | (0.955) |  |
| 50-64 *Death of a relative or friend due to Coronavirus |  |  |  |  |  |  |  |  | -2.275** |  |
|  |  |  |  |  |  |  |  |  | (0.957) |  |
| 65+ * Death of a relative or friend due to Coronavirus |  |  |  |  |  |  |  |  | -1.125 |  |
|  |  |  |  |  |  |  |  |  | (0.959) |  |
| Female* 26-35 (ref. 18-25) * Death of a relative or friend due to Coronavirus |  |  |  |  |  |  |  |  | 0.441 |  |
|  |  |  |  |  |  |  |  |  | (1.500) |  |
| Female* 36-49 * Death of a relative or friend due to Coronavirus |  |  |  |  |  |  |  |  | 1.170 |  |
|  |  |  |  |  |  |  |  |  | (1.322) |  |
| Female* 50-64 * Death of a relative or friend due to Coronavirus |  |  |  |  |  |  |  |  | 2.285* |  |
|  |  |  |  |  |  |  |  |  | (1.233) |  |
| Female* 65+ * Death of a relative or friend due to Coronavirus |  |  |  |  |  |  |  |  | 2.236 |  |
|  |  |  |  |  |  |  |  |  | (1.421) |  |
| Female *A relative or friend was infected |  |  |  |  |  |  |  |  |  | 0.782 |
|  |  |  |  |  |  |  |  |  |  |  |
| Age: 26-35 (ref. 18-25) *A relative or friend was infected |  |  |  |  |  |  |  |  |  | 1.006 |
|  |  |  |  |  |  |  |  |  |  | (0.773) |
| 36-49 *A relative or friend was infected |  |  |  |  |  |  |  |  |  | 0.578 |
|  |  |  |  |  |  |  |  |  |  | (0.700) |
| 50-64 *A relative or friend was infected |  |  |  |  |  |  |  |  |  | 0.304 |
|  |  |  |  |  |  |  |  |  |  | (0.695) |
| 65+ *A relative or friend was infected |  |  |  |  |  |  |  |  |  | -0.0625 |
|  |  |  |  |  |  |  |  |  |  | (0.740) |
| Female* 26-35 (ref. 18-25) *A relative or friend was infected |  |  |  |  |  |  |  |  |  | -1.618 |
|  |  |  |  |  |  |  |  |  |  | (1.031) |
| Female* 36-49 *A relative or friend was infected |  |  |  |  |  |  |  |  |  | -0.446 |
|  |  |  |  |  |  |  |  |  |  | (0.961) |
| Female* 50-64 *A relative or friend was infected |  |  |  |  |  |  |  |  |  | -0.656 |
|  |  |  |  |  |  |  |  |  |  | (0.953) |
| Female* 65+ *A relative or friend was infected |  |  |  |  |  |  |  |  |  | 0.286 |
|  |  |  |  |  |  |  |  |  |  | (1.077) |
| Constant | -1.871** | -1.880** | -1.743** | -1.744** | -1.932** | -1.877** | -1.910** | -2.008*** | -1.978** | -1.839** |
|  | (0.768) | (0.786) | (0.808) | (0.824) | (0.792) | (0.792) | (0.785) | (0.773) | (0.784) | (0.782) |
|  |  |  |  |  |  |  |  |  |  |  |
| Observations | 3,026 | 3,026 | 3,026 | 3,026 | 3,026 | 3,026 | 3,026 | 3,026 | 3,026 | 3,026 |

*Note: N = 3,026. Post-stratification weights are used. *** p<0.01, ** p<0.05, * p<0.1.*

*Source: Intergen-covid online survey. Data were collected between14-24 April 2020*
